# The Work Ahead and How to Accomplish It

**Published:** 1920-01-10

**Authors:** 


### CHRISTMAS AND THE VOLUNTARY HOSPITALS.

### THE WORK AHEAD AND HOW TO ACCOMPLISH IT.

The last few and the present numbers of The Hospital have shown to demonstration the important place filled by the best of our hospitals by their observance of Christmas and the New Year. We have often written, and it is the simple truth, that all who have sympathy with their fellows of all classes, who are Christians in sentiment and conduct, and who accept Christ as their Saviour and Exemplar, find a visit to the hospitals during Christmas to result in benefit and thankfulness where they themselves are concerned, and rejoicing in the evidence they meet with of the whole-hearted, absorbing, and generous personal service rendered by a large number of people of both sexes, young and old, who give their time, their energies, and their abilities to make Christmas memorable and helpful to the inmates of hospital wards.

We commenced our hospital experiences so many years ago when most of the wards at that period were simply white-washed, the bedsteads were mostly of wood, and the Christmas decorations, if there were any, consisted of pieces of holly inserted into the bed-posts by being placed in a hole made with a gimlet by a porter or scrubber told off for the duty. These bits of holly looked so far as our recollection goes forlorn and out of place. They certainly had few attractions, and did not win appreciation for themselves from the patients or visitors, which last were few indeed fifty years ago. To-day in an increasing number of the London hospitals and very many Provincial hospitals Christmas has become a great festival looked forward to by the patients, earnestly prepared for by members of the staff from the top to the bottom, and a time of wholesome fun and rejoicing for the children throughout the country. It is well to recall and remember this, because we hope that Christmas 1919 will be the last which finds the voluntary hospital anywhere in a state of grievous indebtedness and dangerously short of money. In an article on another page we have called attention to the organisation of plans in every hospital centre, whereby it is hoped that the present financial crisis at many a hospital in the United Kingdom may be successfully solved and ended for the future. If this uplifting of grateful hearts and willing hands prove the success we believe it will prove by June 30 next, great blessings must flow from it to all classes of the people, who are for the main part dependent, when ill, upon the voluntary hospital for treatment and the re-establishment of full health, which enables them to resume their ordinary avocations, with pleasure to themselves and profit to their families.

The financial crisis of the hospitals and their future outlook have led us to give special attention this year to Christmas and the methods of keeping this festive season throughout the hospitals of Great Britain. What the hospitals need is the awakening of a large proportion of the ninety per cent. of the population of every centre which has the protection of a well-equipped, up-to-date, and efficient hospital in its midst, to the immense volume of work and the splendour of its nature and results, to the patients who take refuge in its walls, and under Providence, owe their recovery and future prosperity to the benefits they have received there. It is a common experience amongst those who have been at the Front, or have been in close touch with men and women who have been there, that warriors, nurses, and workers of both sexes who have seen and realised for themselves the splendid work done by the hospitals abroad and at home in voluntary hospitals, which have received tens of thousands of wounded men and invalid cases of both sexes arising from the war, have in their hearts the desire to be given an opportunity to testify to their experience in these matters, and their readiness to lend a hand to help the hospitals, now they have returned from the war to their own homes and centres, which they will find at present suffering from a lack of ready money to carry on their work, owing to the heavy liabilities the managers have incurred during the war in providing for the wounded and the sick. Knowing these things from personal experience and direct investigations at the Front and at home, we have been struck with the opportunity Christmas 1919 has afforded the journalist, to call special and particular attention to the life in our voluntary hospitals at home during the season of Christmas to Twelfth Night. During more than fifty years over which our vision and experience extends there has been a revolution in hospital methods—we might say many revolutions, if we include the vast changes and improvements which have taken place in the diagnosis, treatment, and care of the sick, the extended use of ever-improving scientific methods, the growth of technical and practical knowledge, the skill and wonderful diagnostic developments now to be met with in every one of the great hospitals, the gradual disappearance of rough-and-ready treatment, and indifferent consideration for the suffering and trials of patients, the substitution of the best of food and sustenance of all types, the higher standards of knowledge, efficiency, and personal service rendered by the nursing staff, and indeed everything to be met with in the daily

**Christmas and the Voluntary Hospitals**—(*continued*).

routine, and carrying on of the work of a great voluntary hospital of the modern, up-to-date type. All these improvements and developments have necessarily entailed a much greater expenditure of money, but the added cost up to the time of the third year of the war was nobly covered by the generosity of a multitude of givers, of both sexes, in most parts of the United Kingdom, in addition to the splendid finance and other aid rendered through the British Red Cross to all hospitals, where the horrors of war and the needs of the wounded ever increasingly demanded more and more personal service, the extended help of ambulances and all forms of equipment calculated to lessen pain and expedite removal, whilst they secured the maximum of good results for all warriors and other workers at the Front who had fallen and suffered in the war.

When in the fourth and subsequent year the number of wounded warriors and others needing hospital care and the most skilled treatment attained the maximum, the demands upon hospitals everywhere grew to such an extent as to be almost overwhelming. Yet the authorities stuck manfully to their tasks, with the aid of the British Red Cross Organisation and its funds, and succeeded in meeting every call, however terrible or extensive, which the war brought in its train. It is the last two years which have produced such serious financial strain and deficiency in connection with our voluntary hospitals, for the sum involved has been so large as seriously to exceed the amount so liberally supplied year by year by the generous public. These were represented by contributors who collectively did not probably exceed more than one in ten of the whole population of any district where the hospitals which did the work so successfully and well were placed. The grave deficiencies resulting from this state of affairs have yet to be met, and the hope is that by dividing Great Britain into counties and cities it will be possible, with the aid of the personal services of thousands of men and women who are now home from the Front, to organise a canvass and propaganda which will bring home to the majority of the members of every class, which collectively constitute the ninety per cent. who have not yet learnt to give to hospitals, their privilege to do so, that the necessary funds may be forthcoming, and that the actual increased volume of revenue needed by the voluntary hospitals in Great Britain can be organised, so that it may continuously flow, year by year, when the huge war debt referred to has been met and discharged.

If the consciences and knowledge of the majority of men and women throughout Great Britain can be aroused to take a genuine interest in the hospitals, and to base that interest on the knowledge of the work they have done and the indispensable nature of the service rendered, our experience proves that all the money and all the personal service which can be needed will be gladly forthcoming. Having this in mind, we published last week some special articles, bringing out as impressively as we could the realities, the important and far-reaching need of the services and teaching to be met with in Guy's and other hospitals during the Christmas celebrations. We are completing our efforts in this direction by adding a number of illustrations which demonstrate the excellence of the artistic efforts, the inventive genius, and the devoted labours of sisters, residents, and staff in their endeavour to make Christmas in the hospitals a living reality, and to provide that it shall prove not only cheering, but helpful, in the moral and spiritual sense, to the patients, and to all who had the privilege of taking part in the work of the hospitals during this season. Our aim is to make all these improved developments contribute to the uplifting and improvement of the moral and social aspects of the homes of the people. Indeed, nowadays the work of the hospital almoner, the work of the sisters and nurses in the wards, the work done in the massage department, and also in the wounded, out-patient, children's, light, electricity, and many other departments of a great voluntary hospital, has exercised, directly and indirectly, most invaluable influence upon the patients, whose privilege it has been to gain some, or all, of these aids to renewed health and the living of a wiser and better life, brought within their compass. These many victims of human suffering, often hardened by surroundings and circumstances, become materially changed for the better by their residence and treatment in a great hospital.

In the same way the first introduction of men of business, women of all ranks, and even leading men of the day, to the field in which our hospitals carry on their work, has led many of them, from His Majesty the King downwards, to come to regard personal service in the days of health in the cause of the sick as one of the privileges of a hale man, whatever his rank or social position in the country. Therefore we offer no excuse, but merely tender this explanation to the readers of The Hospital, and, through them, to all classes of people in every centre of Great Britain where there is a hospital, that they may of deliberate purpose, for the future, look into this question of the needs and work of our hospitals. They will then be in a position to decide for themselves whether they can, with the knowledge and experience thus obtained, refrain from following His Majesty's example by giving every year a little of their time to the service of the sick through the hospitals wherever they may reside?

The next six months should give everybody everywhere throughout Great Britain, man, woman, aye, and child, a unique opportunity to commence giving personal service in the days of health in the cause of the sick. If this teaching can be brought home through the hospitals and those who use the hospitals everywhere throughout England, Wales, and Scotland, it must have a material influence for good upon the whole population. When this has been attained what is now known as the hospital crisis should be ended and done with for ever.

